# NRF2 Protection against Liver Injury Produced by Various Hepatotoxicants

**DOI:** 10.1155/2013/305861

**Published:** 2013-05-23

**Authors:** Jie Liu, Kai Connie Wu, Yuan-Fu Lu, Edugie Ekuase, Curtis D. Klaassen

**Affiliations:** ^1^University of Kansas Medical Center, Kansas City, KS 66160, USA; ^2^Zunyi Medical College, Zunyi 563003, China

## Abstract

To investigate the role of Nrf2 as a master defense against the hepatotoxicity produced by various chemicals, Nrf2-null, wild-type, Keap1-knock down (Keap1-Kd) and Keap1-hepatocyte knockout (Keap1-HKO) mice were used as a “graded Nrf2 activation” model. Mice were treated with 14 hepatotoxicants at appropriate doses, and blood and liver samples were collected thereafter (6 h to 7 days depending on the hepatotoxicant). Graded activation of Nrf2 offered a Nrf2-dependent protection against the hepatotoxicity produced by carbon tetrachloride, acetaminophen, microcystin, phalloidin, furosemide, cadmium, and lithocholic acid, as evidenced by serum alanine aminotransferase (ALT) activities and by histopathology. Nrf2 activation also offered moderate protection against liver injury produced by ethanol, arsenic, bromobenzene, and allyl alcohol but had no effects on the hepatotoxicity produced by D-galactosamine/endotoxin and the Fas ligand antibody Jo-2. Graded Nrf2 activation reduced the expression of inflammatory genes (MIP-2, mKC, IL-1**β**, IL-6, and TNF**α**), oxidative stress genes (Ho-1, Egr1), ER stress genes (Gadd45 and Gadd153), and genes encoding cell death (Noxa, Bax, Bad, and caspase3). Thus, this study demonstrates that Nrf2 prevents the liver from many, but not all, hepatotoxicants. The Nrf2-mediated protection is accompanied by induction of antioxidant genes, suppression of inflammatory responses, and attenuation of oxidative stress.

## 1. Introduction

Nuclear factor erythroid 2-related factor 2 (Nrf2) is a transcription factor that promotes transcription of a battery of cytoprotective genes in response to oxidative/electrophilic stress [1]. Under basal conditions, Nrf2 is sequestered by kelch-like ECH associating protein 1 (Keap1) in the cytosol. In response to oxidative stress, Nrf2 is released from Keap1, translocates into the nucleus, and induces an array of cytoprotective genes as adaptive responses [1, 2]. Nrf2 target genes include NAD(P)H quinone oxidoreductase 1 (Nqo1), GSH synthesis (Gclc and Gclm), GSH conjugation (Gsts), and many other oxidized protein repair genes [2, 3]. A “graded Nrf2 activation” animal model, consisting of Nrf2-null mice, wild-type mice, Keap1-knockdown (Keap1-KD) mice with enhanced Nrf2 activation, and Keap1-hepatocyte knockout (Keap1-HKO) mice with maximum Nrf2 activation, has been used to study the functions of Nrf2 in the liver [3, 4]. Transcription profiling of the “graded Nrf2 activation” animal model by microarray analysis showed that many cytoprotective genes are constitutively expressed in a “gene dose-response” manner [3–5].

Using the “graded Nrf2 activation” animal model, this laboratory has reported that Nrf2 protects against the hepatotoxicity produced by cadmium [5], ethanol [6], and diquat [7]. In addition to the “graded Nrf2 activation,” Nrf2-null mice are more sensitive to the hepatotoxicity produced by acetaminophen [8–10], 1-bromopropane [11], and the chronic hepatotoxicity produced by carbon tetrachloride [12], arsenic [13], and fatty liver from feeding a methionine- and choline-deficient (MCD) diet [14]. However, graded Nrf2 activation did not confer protection against the steatosis from feeding a high fat diet for 6 months [15]. 

The purpose of the present study was to use this unique “graded Nrf2 activation” animal model to determine whether various levels of basal expression of Nrf2 protect against acute liver injury produced by 14 hepatotoxicants. Each of these hepatotoxicants produces liver injury by various mechanisms, which will provide further insight into the mechanism of how Nrf2 protects against hepatotoxicants. This study focuses on inflammatory responses and oxidative stress as potential mechanisms of Nrf2-mediated hepatoprotection.

## 2. Results

### 2.1. Graded Nrf2 Activation in a Genetic Animal Model

To verify the graded Nrf2 activation, the expression of Nrf2 and Nrf2-targeted genes was quantified in 4 genotypes of mice.  Table 1 shows that the higher basal expression of the cytoprotective genes in Keap1-KD and Keap1-HKO mice, for example, the expression of Nqo1 (0.037 for Nrf2, 0.293 for WT, 0.624 for Keap1-KD, and 3.17 for Keap1-HKO, % of G3PDH) and Gclc (3.84 for Nrf2, 7.02 for WT, 9.06 for Keap1-KD, and 21.4 for Keap1-HKO, % of G3PDH) was markedly higher in Keap1-HKO mice as compared to WT mice. Similarly, the expression of GSH homeostasis and conjugation genes such as glutathione reductase (Gsr), glutathione *S*-transferase (GSTmu, GSTa1, GSTa4), and glutathione peroxidase (Gpx2) was also increased in a “gene-dose” manner. In addition, the basal expression of heme oxygenase-1 (Ho-1) and metallothionein (Mt-1), two other cytoprotective mechanisms [16, 17], was also higher in Keap1-HKO mice, confirming prior publications [4, 5]. 

### 2.2. Effects of Nrf2 in Protection against 14 Hepatotoxicants

Table 2 shows the degree of liver injury produced by various hepatotoxicants, based on increased serum enzyme activities of alanine aminotransferase (ALT) and on histopathology. Nrf2-null mice were highly susceptible to the hepatotoxicity produced by CCl_4_, acetaminophen, microcystin, phalloidin, furosemide, cadmium [5], and lithocholic acid. Graded activation of Nrf2 offered protection in a “gene-dose” manner. The hepatotoxicity produced by ethanol [6], diquat [7], and arsenic was mild, and the hepatotoxicity produced by bromobenzene and allyl alcohol was moderate, and the Nrf2-null mice were more susceptible than Keap1-KD or Keap1-HKO mice to liver injury. In comparison, graded Nrf2-activation did not offer protection against the hepatotoxicity produced by D-galactosamine/endotoxin and the Fas ligand antibody Jo-2 (Table 2).

#### 2.2.1. Liver Histopathology

**Figure 1:**
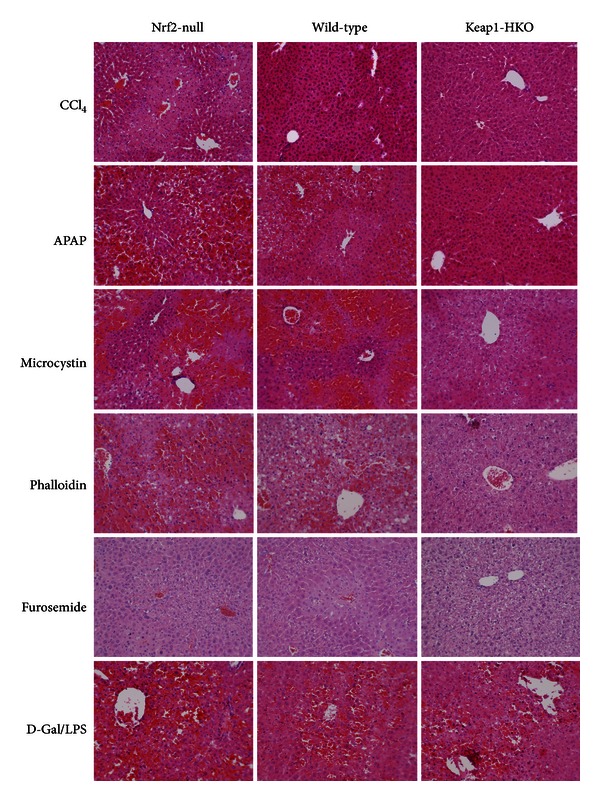
Histological analysis of livers from Nrf2-null, wild-type, and Keap1-HKO mice treated with CCl_4_ (25 *μ*L/kg, ip, 16 h), acetaminophen (APAP, 400 mg/kg, ip for 8 h), microcystin (50 *μ*g/kg, ip, 8 h), phalloidin (1.5 mg/kg, ip, 8 h), furosemide (250 mg/kg, ip 24 h), and D-galactosamine/endotoxin (400 mg/10 *μ*g/kg, ip, 8 h). Arrows indicate hemorrhage and inflammation and arrowheads indicate hepatocellular necrosis (200x).

There were no observable abnormalities in livers of control Nrf2-null, wild-type, and Keap1-HKO mice (data not shown).  Figure 1 shows representative histopathology after 6 hepatotoxicants in the “graded Nrf2 activation” animal model. Microcystin, phalloidin, and acetaminophen produced severe hemorrhage and inflammation in Nrf2-null mice, but these pathological lesions were greatly attenuated in Keap1-HKO mice. Extensive necrosis was produced by CCl_4_, acetaminophen, microcystin, and furosemide, but only mild hepatocyte swelling with no apparent necrosis was evident in Keap1-HKO mice (Figure 1). Furosemide produced foci of inflammation and necrosis in Keap1-HKO mice, which are more severe in Nrf2-null mice. However, graded Nrf2 activation did not show difference in the histopathology produced by D-galactosame/endotoxin (Figure 1) and the Fas ligand antibody Jo-2 (data not shown).

### 2.3. Effects of Nrf2 Activation on Inflammation and Stress Gene Expression

#### 2.3.1. Expression of Chemokine Genes

The mRNA expression of neutrophil-specific chemokine macrophage inflammatory protein 2 (MIP-2) and mouse keratinocyte-derived chemokine (mKC) is shown in Table 3. There was no difference in basal expression of MIP-2 (around 0.045% of G3PDH) and mKC (around 2% of G3PDH) among the four genotypes (data not shown). After hepatotoxicant challenge (microcystin, phalloidin, and lithocholic acid), mRNA levels of MIP-2 (20–30 fold) and mKC (5–15 fold) were increased in Nrf2-null mice, which were greatly attenuated in Keap1-HKO mice, indicating that Nrf2-overexpression attenuated toxicant-induced inflammatory response in a “graded Nrf2 activation” manner.

#### 2.3.2. Expression of Inflammation Genes

mRNA levels of proinflammatory genes interleukin-1*β* (IL-1*β*), IL-6, and TNF*α* showed a similar pattern (Table 3). There was no difference in basal expression of IL-1*β* (around 0.026% of G3PDH), IL-6 (around 0.002% of G3PDH), and TNF*α* (around 0.004% of G3PDH) among the four genotypes (data not shown). Toxicants (microcystin, phalloidin, acetaminophen, CCl_4_, and lithocholic acid) administration markedly increased mRNA levels of these inflammatory cytokines in Nrf2-null mice, but only mild increases were seen in Keap1-HKO mice, indicating that Nrf2-overexpression attenuated toxicant-induced hepatic inflammation.

#### 2.3.3. Expression of Acute Phase Protein Genes

mRNA levels of acute phase protein genes heme oxygenase-1 (Ho-1) and early growth response gene-1 (Egr-1) showed a similar pattern (Table 3). The basal levels of Ho-1 were higher in Keap1-HKO mice (Table 1) [6], and after hepatotoxicant insults, more increases in Ho-1 were observed in Nrf2-null mice than in Keap1-HKO mice; a similar pattern holds true for Egr-1, but the basal levels of Egr-1 were lower in Keap1-HKO mice. Acetaminophen, CCl_4_, microcystin, phalloidin, and lithocholic acid are all effective inducers of the mRNA of two acute phase protein genes Ho-1 and Egr-1, implying higher levels of stress in the Nrf2-null mouse livers. 

### 2.4. Effects of Nrf2 Activation on ER Stress and Cell-Death Genes

#### 2.4.1. Expression of Genes Involved in ER Stress

The mRNA levels of two proteins involved in endoplasmic reticulum (ER) stress are shown in Table 4. After the challenge by CCl_4_, acetaminophen, microcystin, and phalloidin, higher expression of Gadd45 and Gadd153 was observed in Nrf2-null mice, moderate in wild-type mice and lower expressed in Keap1-KD and Keap1-HKO mice, respectively, indicating the “graded Nrf2 activation” dose dependently protected against toxicant-induced ER stress. 

#### 2.4.2. Expression of Genes Involved in Cell Death

The mRNA levels of genes involved in cell death are shown in Table 4. Cell death-related genes such as Noxa, Bax, Bad, and caspase3 encode cell apoptosis and cell death pathways. After toxicant insults, (CCl_4_, acetaminophen, microcystin, phalloidin, etc.), higher expression of these genes was seen in Nrf2-null mice, but only mild increases were evident in the livers of Keap1-KD and Keap1-HKO mice, indicating the protection against cell death with graded Nrf2 activation.

## 3. Discussion

The “graded Nrf2 activation” model [3–6] is a unique animal model to evaluate the role of genetic activation of Nrf2 as a host master defense against various hepatotoxicants. The present study indicates that the genetic activation of Nrf2 results in mice more resistant to the acute hepatotoxicity produced by CCl_4_, acetaminophen, microcystin, phalloidin, cadmium, furosemide, and lithocholic acid in a “Nrf2 gene-dose” manner. Nrf2 activation also offered moderate protection against the hepatotoxicity produced by ethanol [5] and arsenic. However, overexpression of Nrf2 had limited effects on the hepatotoxicity of D-galactosamine/endotoxin and the Fas ligand antibody Jo-2. Thus, activation of Nrf2 offered protection against many, but not all, hepatotoxicants.

The hepatotoxicants used in the present study produce liver injury through different mechanisms. Some hepatotoxicants require metabolic activation to produce reactive intermediates such as the reactive intermediate *N*-acetyl-*p*benzoquinoneimine (NAPQI) produced by acetaminophen [18] and trichloromethyl radical (CCl_3_^∙^) produced by carbon tetrachloride [19]. The hepatotoxicity of bromobenzene derives from its reactive metabolites (epoxides and quinones), which arylate cellular proteins [20]. Cadmium and arsenic are metallic toxicants not requiring bioactivation to produce reactive intermediate, rather by producing oxidative stress in their acute toxicity [16, 21, 22]. Redox-cycling metabolism of the bipyridilium herbicide, and diquat generates oxidative stress which results in cytotoxicity and liver injury [23]. Acute microcystin poisoning is characterized by inhibition of serine/threonine phosphatases and subsequent hyperphosphorylation of cytoskeletal proteins, leading to disruption of hepatocyte architecture [24]. Phalloidin binds to F-actin, preventing trafficking along the cytoskeleton and “freezing” hepatocytes [25]. D-galactosamine/LPS and the Fas ligand Jo2 antibody treatment are well-established models of liver injury mediated by innate immunity [26, 27]. “Graded Nrf2 activation” seems to offer a generalized hepatoprotection against most hepatotoxicants under investigation except for D-galactosamine/LPS and the Fas ligand Jo2 antibody. Thus, the expression of genes related to hepatotoxicity was performed to look into the generalized mechanism.

The neutrophil-specific chemokine macrophage inflammatory protein 2 (MIP-2, CXCR2) and mouse keratinocyte-derived chemokine (mKC) are important mediators of inflammation in acute tissue injury [28]. In liver inflammation, recruitment of circulating polymorphonuclear leukocytes is essential for host defense and initiates specific immune responses. One pathological hallmark of acute liver injury is the uncontrolled transmigration of neutrophils into the liver. The extravasation of leukocytes from the vascular system into the liver is induced by chemokines that are released from the site of inflammation. In the present study, toxicant-elevated MIP-2 and mKC were significantly reduced in Keap1-HKO mice, indicating that activation of Nrf2 might reduce toxicant-induced liver injury which is mediated in part by reducing liver inflammation, or that with less toxicity there was less detection of the inflammatory cytokines. Proinflammatory cytokines such as tumor necrosis factor alpha (TNF*α*), interleukin 1beta (IL-1*β*), and interleukin 6 (IL-6) play important roles in acute liver damage [29]. Thus, these proinflammatory cytokine increases are implicated in toxicant-induced liver injury and can be detected at the molecular level. Again, Keap1-HKO mice had much lower expression of these cytokines, indicating that activation of Nrf2 reduces toxicant-induced liver injury in part by reducing liver proinflammatory cytokine release.

Inflammation is often associated with overproduction of reactive oxygen species (ROS) that play an important role in toxicant-induced acute liver injury. The increased lipid peroxidation is a sensitive biomarker for the hepatotoxicity produced by CCl_4_ [30], acetaminophen [31], microcystin [32], cadmium [6, 16], ethanol [5], and diquat [7]. In combating against increased oxidative stress, the GSH synthesis and conjugating enzyme genes are increased as a Nrf2-targeted host defense against oxidative stress in Keap1-HKO mice in the protection against the hepatotoxicity produced by cadmium [5, 6], microcystin [33], and acetaminophen [31]. Thus, the reduction of oxidative stress, probably through the enhancement of the GSH system is one of the important mechanisms for Nrf2-mediated protection against hepatotoxicity.

Acute phase proteins are important adaptive mechanism in response to acute stress. For example, early growth response (Egr)-1, a transcription factor that regulates expression of inflammatory genes, plays a pathological role in many animal models of acute and chronic inflammatory disease [34]. Heme oxygenase-1 (Ho-1) is an essential enzyme which degrades heme into carbon monoxide, biliverdin, and free iron. Induction of Ho-1 in rodent models of acute and chronic hepatic inflammation results in improvement of liver damage and downregulation of proinflammatory cytokines [17]. Ho-1 is a Nrf2-targeted gene, and it is higher in Keap1-HKO mice under basal conditions. The higher the oxidative stress, the higher expression of Ho-1, and thus Ho-1 is also a biomarker for oxidative stress [5, 17]. Metallothioneins are important for cadmium detoxication [16]. Higher induction of these acute phase protein genes was observed in Nrf2-null mice, as compared to Keap1-HKO mice, implying higher generation of ROS and stress from toxicants in mice deficient in Nrf2. 

Endoplasmic reticulum (ER) is the site of synthesis and folding of proteins. Perturbations of ER homeostasis affect protein folding and cause ER stress. ER stress is implicated in chemical-induced hepatotoxicity. One of the components of the ER stress-mediated apoptosis pathway is C/EBP homologous protein (CHOP), also known as growth arrest- and DNA damage-inducible gene 153 (GADD153) [35]. The Gadd45 stands at the crossroad of cell fate by controlling the balance between cellular DNA repair, eliminating (apoptosis) or preventing the expansion of potentially dangerous cells [36]. As a biomarker of ER stress and DNA damage, both Gadd153 and Gadd45 were markedly increased in Nrf2-null mice as compared to graded Nrf2 activation in the “graded Nrf2 activation” models (Table 4), implying that Nrf2-deficiency makes animals susceptible to ER stress.

Lesions to DNA trigger the DNA-damage response, a complex, multibranched cell-intrinsic process targeted to DNA repair or elimination of damaged cells by apoptosis [37]. The Bcl2 homology domain 3 (BH3)-only protein Noxa is at the tip of the balance between life and death and appears to be crucial for cell death along the mitochondrial Bcl2-regulated apoptosis pathway in response to toxicant insults, presumably by sensitizing the cell toward the action of additional BH3-only protein family members [38]. BAX, the BCL-2-associated X protein, is a cardinal proapoptotic member of the BCL-2 family. BAD, a BH3-only pro-apoptotic protein, helps coordinate mitochondrial fuel metabolism and the apoptotic machinery. Both regulate the critical balance between cellular life and death [39, 40]. Death-mediating proteases, caspase-3 in particular, have been implicated in as a bifurcation point between plasticity and cell death [41]. In the present study, mice deficient in Nrf2 were highly susceptible to toxicant-induced apoptosis and necrosis, with marked upregulation of these proapoptotic components. In comparison, Nrf2 activation reduced cell death as well as the mRNA levels of these apoptosis executors. 

However, graded activation of Nrf2 does not confer protection against the hepatotoxicity produced by D-galactosamine/endotoxin and the Fas ligand antibody Jo-2 (Table 2). Both hepatotoxicants produce liver injury through the modulation of the immune system. Little is known about the effect of Nrf2 on endotoxemia and immune modulation. Keap1-KD mice and CDDO-Im-induction of Nrf2 are effective in decreasing concanavalin A-induced liver injury and the late-phase proinflammatory gene expression in the liver [42]. Our recent work shows that activation of Nrf2 suppresses IFN-*γ* production, while inducing the production of the Th2 cytokines IL-4, IL-5, and IL-13. Nrf2 activation also suppresses T-bet DNA binding and promotes GATA-binding protein 3 DNA binding [43], suggesting that Nrf2 activation skews CD4(+) T cells toward Th2 differentiation and, thus, represents a novel regulatory mechanism in CD4(+) T cells. The role of Nrf2 activation in immune functions requires further investigation. 

In conclusion, the present study shows that “graded Nrf2 activation” markedly decreased the hepatotoxicity of most hepatotoxicants. The protective effect of Nrf2 is accompanied by induction of genes involved in antioxidant defense, and genes involved in host defense against toxicity stimuli.

## 4. Materials and Methods

### 4.1. Reagents

Carbon tetrachloride (CCl_4_), acetaminophen (APAP), microcystin (Microcystin-LA), phalloidin, lithocholic acid (LCA), sodium arsenite (As3+), D-galactosamine, lipopolysaccharide (LPS), furosemide, allyl alcohol, and bromobenzene were purchased from Sigma-Aldrich (St. Louis, MO, USA). The Fas ligand antibody Jo-2 was obtained from BD Biosciences (San Jose, CA, USA). All other chemicals were reagent grade and commercially available.

### 4.2. Animal Husbandry and Treatment

C57BL/6 breeders were purchased from Charles River Laboratories, Inc. (Wilmington, MA, USA). Eight-week-old male mice were used for this study. Nrf2-null mice were obtained from Dr. Jefferson Chan (University of California, Irvine, CA, USA) [44]. Keap1-KD mice were supplied by Dr. Masayuki Yamamoto (Tohoku University, Sendai, Japan). In an attempt to make a hepatocyte-specific Keap1-null mouse, utilizing a loxP, Alb-Cre system, a Keap1-KD mouse was engineered, in which Keap1 was decreased throughout the body [45]. Nrf2-null mice were backcrossed into the C57BL/6 background, and >99% congenicity was confirmed by Jackson Laboratories (Bar Harbor, ME, USA). Keap1-HKO mice were generated by crossing Keap1-KD mice and AlbCre^+^ mice, which express Cre only in hepatocytes. All the mice were bred at the University of Kansas Medical Center, housed in a temperature-, light-, and humidity-controlled environment, and had access to Teklad Rodent Diet #8604 (Harlan Laboratories, Madison, WI, USA) and water *ad libitum*. The housing facility is accredited by the Association for Assessment and Accreditation of Laboratory Animal Care. The animal treatment protocols were approved by the University of Kansas Medical Center Institutional Animal Care and Use Committee.

### 4.3. Experimental Design

Nrf2-null mice, wild-type mice, and Keap1-HKO mice were treated with acetaminophen (500 mg/kg, ip 8 h), carbon tetrachloride (25 *μ*L/kg, 16 h), microcystin (50 *μ*g/kg, i.p., 8 h), phalloidin (1.5 mg/kg, ip, 8 h), lithocholic acid (0.4% in diet for 7 days), sodium arsenite (13 mg/kg, i.p., 24 h), or saline (10 mL/kg, i.p.), D-galactosamine/LPS (400 mg/10 *μ*g/kg, i.p., 8 h), furosemide (250 mg/kg, ip 24 h), bromobenzene (0.7 mL/kg), allyl alcohol (85 mg/kg, ip, 24 h), and the Fas ligand Jo-2 (5 *μ*g/mouse). At the end of the experiments, mice were anesthetized with pentobarbital (50 mg/kg, ip). Blood and liver samples were collected. Portions of livers were fixed in 10% neutral formalin for histological analysis, and others were frozen in liquid nitrogen and stored at −80°C. The dose selection is based on our previous publications [46] and pilot experiments.

### 4.4. Hepatotoxicity Evaluation

Serum alanine aminotransferase (ALT) and aspartate aminotransferase (AST) activities were determined as a biochemical indicator of hepatocellular necrosis using Pointe Scientific Liquid ALT and AST Reagent (Canton, MI, USA) according to the manufacturer's protocol.

### 4.5. Histopathology

Liver samples were fixed in 10% formalin prior to routine processing and paraffin embedding. Liver sections (5 *μ*m in thickness) were stained with hematoxylin and eosin and evaluated for hepatocellular necrosis.

### 4.6. Total RNA Isolation

Total RNA was isolated using RNAzol B reagent (Tel-Test, Inc., Friendswood, TX, USA) according to the manufacturer's protocol. The concentration of total RNA in each sample was quantified spectrophotometrically at 260 nm. The integrity of each RNA sample was evaluated by formaldehyde-agarose gel electrophoresis before analysis.

### 4.7. Quantification of mRNA by RT-PCR

Total RNA in mouse livers was reverse-transcribed into cDNA by High Capacity cDNA Archive Kit (Applied Biosystems, Foster City, CA, USA), and the resulting cDNA was used for real-time PCR analysis using Power SYBR Green PCR Master Mix in a 7900HT Fast Real-Time PCR System (Applied Biosystems, Foster City, CA, USA). Oligonucleotide primers were designed with Primer3 software and listed in Supplemental Table  1 (available online at http://dx.doi.org/10.1155/2013/305861).

### 4.8. Statistical Analysis

Data were expressed as mean ± SEM and analyzed using a one-way ANOVA followed by Duncan's multiple range test utilizing SPSS 13 Software (SAS, NC). The significant level was set at *P* ≤ 0.05. 

## Supplementary Material

The primer sequence of the interested genes under investigation.

## Figures and Tables

**Table 1:** The basal levels of the Nrf2 and Nrf2-targeted genes in the “graded Nrf2 activation” model.

	Nrf2-null	Wild-type	Keap1-Kd	Keap1-HKO
Nrf2-related				
Nqo1	0.037 ± 0.007*	0.29 ± 0.02	0.74 ± 0.12*	3.17 ± 0.36*
Nrf2	0.002 ± 0.001*	0.07 ± 0.02	1.46 ± 0.05*	2.10 ± 0.24*
Gclc	3.84 ± 0.86*	7.01 ± 1.11	10.1 ± 1.37*	27.2 ± 2.49*
GSH-related				
Gsr	0.11 ± 0.01*	0.21 ± 0.01	0.03 ± 0.01	0.06 ± 0.01*
Gsta1	0.45 ± 0.11*	2.77 ± 1.43	15.4 ± 3.58*	51.1 ± 7.25*
Gsta4	1.48 ± 0.39*	2.97 ± 1.14	4.63 ± 1.02	7.93 ± 1.55*
Gstmu	20.7 ± 1.43*	107 ± 28.3	544 ± 98.2*	1547 ± 350*
Gstpi	101 ± 22.2	7.01 ± 1.11	10.1 ± 1.37*	27.2 ± 2.49*
Gpx2	0.02 ± 0.01	0.04 ± 0.03	0.06 ± 0.01	2.15 ± 0.81*
Acute-phase				
Mt-1	4.22 ± 0.93	3.48 ± 1.11	20.1 ± 5.43*	27.4 ± 2.49*
Ho-1	0.46 ± 0.06	0.64 ± 0.03	0.87 ± 0.15*	1.23 ± 0.37*

Data are % of the housekeeping gene G3PDH and represent mean ± SEM of serum ALT values (*n* = 6–10), *significantly different from wild-type mice, *P* < 0.05.

**Table 2:** The “graded Nrf2 activation” model in protecting against hepatotoxicants.

Hepatotoxicants	Dose, route, and time	Nrf2-null	Wild-type	Keap1-Kd	Keap1-HKO	References
Carbon tetrachloride	25 *μ*l/kg, ip, 16 h	4610 ± 920	2970 ± 690	2110 ± 464	503 ± 301*	Unpublished
Acetaminophen	500 mg/kg, ip, 8 h	1010 ± 385	615 ± 165	266 ± 93*	198 ± 43*	Unpublished
Microcystin	50 *μ*g/kg, ip, 8 h	1010 ± 385	615 ± 165	266 ± 93*	198 ± 43*	Unpublished
Phalloidin	1.5 mg/kg, ip, 8 h	4130 ± 705	3630 ± 1185	1410 ± 348	210 ± 87*	Unpublished
Furosemide	250 mg/kg, ip, 18 h	1095 ± 365	570 ± 393	40 ± 8*	35 ± 5*	Unpublished
Cadmium	3.5 mg/kg, ip, 8 h	995 ± 152	675 ± 98	201 ± 21*	111 ± 15*	Wu et al., 2012a
Arsenic	100 umol/kg, ip, 8 h	165 ± 52	120 ± 53	66 ± 29	52 ± 18*	Unpublished
Ethanol	5 g/kg, po, 6 h	70 ± 20	88 ± 25	46 ± 11	38 ± 5*	Wu et al., 2012b
Diquat	125 mg/kg, ip, 6 h	604 ± 65	412 ± 48	302 ± 16*	N.D.	Wu et al., 2012c
Bromobenzene	0.7 ml/kg, ip, 24 h	1650 ± 235	1550 ± 211	N.D.	175 ± 35*	Unpublished
Allyl Alcohol	85 mg/kg, ip, 24 h	1130 ± 211	1050 ± 330	N.D.	220 ± 65*	Unpublished
Lithocholic acid	0.4% in diet, 7 d	1390 ± 216	985 ± 211	450 ± 112	105 ± 25*	Unpublished
D-Gal/LPS	400 mg/10 ug/kg, ip, 6 h	4630 ± 1250	3730 ± 660	5430 ± 1005	3650 ± 535	Unpublished
Fas Jo2 antibody	5 *μ*g/mouse, ip, 8 h	4815 ± 1310	5530 ± 2105	5545 ± 1950	6170 ± 2510	Unpublished

Data represent mean ± SEM of serum ALT values (*n* = 6–10), *significantly different from wild-type mice, *P* < 0.05. N.D.: not detected.

**Table 3:** Inflammatory gene expressions under the challenge of hepatotoxicants.

	Nrf2-null	Wild-type	Keap1-Kd	Keap1-HKO
MIP-2	+++	++	+	±
mKC	+++	++	+	±
ICAM1	++	++	+	+
IL-1*β*	+++	++	+	±
IL-6	++	+	±	±
TNF*α*	++	+	±	±
Egr1	++	++	+	+
Ho-1	+++	++	+	+

MIP-2: macrophage inflammatory protein 2; mKC: mouse keratinocyte-derived chemokine; IL-1*β*: interleukin-1*β*; IL-6: interleukin-6; TNF*α*: tumor necrosis factor *α*. Ho-1: heme oxygenase-1. “+++” indicates significant increase; “++” indicates moderate increase; “+” indicates slight mild increase; “±” indicates slight increase. These inflammatory gene expression profiles are similar for the hepatotoxicity produced by acetaminophen, carbon tetrachloride, microcystin, phalloidin, and furosemide.

**Table 4:** Expression of DNA damage genes and genes of cell death.

	Nrf2-null	Wild-type	Keap1-Kd	Keap1-HKO
DNA damage				
Gadd45	++++	+++	++	+
Gadd153	++++	+++	++	+
Apoptosis				
Noxa	+++	++	+	+
Bax	++	++	+	+
Bad	++	++	+	+
Casp3	++	++	+	+

Gadd45: the growth arrest and DNA damage-inducible 45 proteins; Gadd153/Chop10: C/EBP homologous protein 10; Noxa: a BH3-only member of the Bcl-2 family and candidate mediator of p53-induced apoptosis; Bax: Bcl-2 associated protein X; Bad: BCL2-associated agonist of cell death; Casp3: caspases 3. “+++” indicates significant increase; “++” indicates moderate increase; “+” indicates slight mild increase. These inflammatory gene expression profiles are similar for the hepatotoxicity produced by acetaminophen, carbon tetrachloride, microcystin, phalloidin, and furosemide.
