# Supplementary material for: NRF2 Protection against Liver Injury Produced by Various Hepatotoxicants
Source: Oxid Med Cell Longev. 2013 May 23;2013:305861. doi: 10.1155/2013/305861 (PMC3676920; doi:10.1155/2013/305861)
Supplement: Supplementary file 1 — The primer sequence of the interested genes under investigation. [file 305861.f1.docx]

Supplementary Table 1:

Primer sequences for real-time RT-PCR analysis

| Gene | GenBank# | Forward | Reverse |
| --- | --- | --- | --- |
| Casp3 | NM_009810 | GGGCGTGTTTCTGTTTTGTT | TTGAGGTAGCTGCACTGTGG |
| Bad | NM_007522 | CTCCACATCCCGGAACTCTA | TTAAAGGGACACAGCGATCC |
| Bax | NM_007527 | AGGCCTCCTCTCCTACTTCG | CTCAGCCCATCTTCTTCCAG |
| Egr1 | M20157 | AGGTTCCCATGACCCTGACT | GGTACGTTCTCCAGACCCTG |
| G3PDH | M32599 | AACTTTGGCATTGTGGAAGG | GGATGCAGGGATGATGTTCT |
| Gclc | BC019374 | TGGCCACTATCTGCCCAATT | GTCTGACACGTAGCCTCGGTAA |
| Gadd45 | NM_007836 | TGAGCTGCTGCTACTGGAGA | TCCCGGCAAAAACAAATAAG |
| Gadd153 | X67083 | CTGCACCAAGCATGAACAGT | CTACCCTCAGTCCCCTCCTC |
| Gpx2 | NM_030677 | CAGCTTCCAGACCATCAACA | CACTGAGCCCTGAGGAAGAC |
| Gsr | NM_010344 | CGGCCACTCCACTCATTATT | AACGTGCAGGTTTTGTTTCC |
| Gsta1 | NM_008181 | CGCCACCAAATATGACCTCT | TTGCCCAATCATTTCAGTCA |
| Gsta4 | AK008490 | CTATGTTGAGGTGGTCAGGACTGT | CTGTGGTGACACTGCAATTGG |
| Gstm1 | NM_010358 | CTCCCGACTTTGACAGAAGC | TTGCTCTGGGTGATCTTGTG |
| Gstpi | D30687 | TGGGCATCTGAAGCCTTTTG | GATCTGGTCACCCACGATGAA |
| Ho-1 | M33203 | CCTCACTGGCAGGAAATCATC | CCTCGTGGAGACGCTTTACATA |
| ICAM1 | NM_010493 | GTCTCGGAAGGGAGCCAAGTA | CGACGCCGCTCAGAAGAA |
| IL-1β | NM_008361 | CTGGTGTGTGACGTTCCCATTA | CCGACAGCACGAGGCTTT |
| IL-6 | J03783 | GCCCACCAAGAACGATAGTCA | GAAGGCAACTGGATGGAAGTCT |
| MIP2 | NM_009140 | CCTCAACGGAAGAACCAAAGAG | CTCAGACAGCGAGGCACATC |
| mKC | NM_008176 | TGGCTGGGATTCACCTCAAG | GTGGCTATGACTTCGGTTTGG |
| Noxa | AB041230 | CCCACTCCTGGGAAAGTACA | AATCCCTTCAGCCCTTGATT |
| Mt-1 | NM_013602 | CTCCGTAGCTCCAGCTTCAC | AGGAGCAGCAGCTCTTCTTG |
| Nqo1 | BC004579 | TATCCTTCCGAGTCATCTCTAGCA | TCTGCAGCTTCCAGCTTCTTG |
| Nrf2 | BC026943 | CGAGATATACGCAGGAGAGGTAAGA | GCTCGACAATGTTCTCCAGCTT |
| TNFα | U68415 | TTTTCCGAGGGTTGAATGAG | CTGGCTAGTCCCTTGCTGTC |
